# Correction: Self-reported impact of the COVID-19 pandemic, affective responding, and subjective well-being: A Swedish survey

**DOI:** 10.1371/journal.pone.0320506

**Published:** 2025-03-17

**Authors:** Maria Gröndal, Karl Ask, Timothy J. Luke, Stefan Winblad

The Data Availability statement for this paper is incorrect. The correct statement is: All relevant data files are available from the OSF database at the following URL: https://osf.io/f8y3n/.

In the Procedure subsection of the Methods, there is an error in the second sentence of the paragraph. The correct sentence is: That study was approved by the Head of the Department of Psychology, University of Gothenburg and the preregistration is available at (https://osf.io/f8y3n/).

In the Procedure subsection of the Methods, the sixth sentence should have been excluded from the paragraph.

In the Measure subsection of the Methods, there is an error in the second sentence of the paragraph. The correct sentence is: The full questionnaire can be found at (https://osf.io/f8y3n/).

In the “How do self-reported impacts of COVID-19 relate to demographics?” subsection of Results, there is an error in the fifth sentence of the paragraph. The correct sentence is: For more complete information, including all nonsignificant results, see the online supplemental materials (https://osf.io/f8y3n/).

## References

[1] Gröndal M, Ask K, Luke TJ, Winblad S. Self-reported impact of the COVID-19 pandemic, affective responding, and subjective well-being: A Swedish survey. PLoS One. 2021;16(10):e0258778. doi: 10.1371/journal.pone.0258778 34653222 PMC8519461

