# Supplementary material for: Self-reported impact of the COVID-19 pandemic, affective responding, and subjective well-being: A Swedish survey
Source: PLoS One. 2021 Oct 15;16(10):e0258778. doi: 10.1371/journal.pone.0258778 (PMC8519461; doi:10.1371/journal.pone.0258778)
Supplement: Attachment — Submitted filename: Response to reviewers.docx [file pone.0258778.s002.docx]

Dear professor Capraro,

**Editor comments**

1. **Please review your reference list to ensure that it is complete and correct. If you have cited papers that have been retracted, please include the rationale for doing so in the manuscript text, or remove these references and replace them with relevant current references. Any changes to the reference list should be mentioned in the rebuttal letter that accompanies your revised manuscript. If you need to cite a retracted article, indicate the article’s retracted status in the References list and also include a citation and full reference for the retraction notice.**

   We have reviewed the reference list to make sure that it is complete and correct. Moreover, we have found that none of the entries in the list have been retracted.

**Reviewer #2 comments**

1. **p. 17; l. 351-259: The Authors may introduce names of Factor 1 and 2 of the self-reported COVID-19 impact. It seems that Factor 1 refers to self-reported COVID-19 impact on free time activities, while the factor 2 refers to self-reported COVID-19 impact on work and family life. The analysis left also unanswered questions about the moderating role of ERQ strategies for associations between irritability and self-reported COVID-19 impact. It could be mentioned in the future analyses that individuals with some personality factors that may have been predictive for experiencing grater burden of the pandemic (e.g. irritability) may be protected by efficient emotion regulation.**

It is only one section of the results where the labels Factor 1 and Factor 2 are used as predictors in models. As such, we don’t think it’s worth introducing a name for these indexes, both because of the brevity of that section and also to avoid the naming fallacy. However, we agree that some additional clarity about these factors may be useful, so we have added some parentheticals to the section to make it especially clear what items the factors comprise. Moreover, in the Discussion, we refer to the factors in terms of their actual content (e.g., “The reported impact of the pandemic on family life, work/study, and finances…”) rather than factor numbering.

We are hesitant to add any further analyses to the paper, as it already contains a considerable amount of results. However, in response to this comment, we examined the possibility that irritability interacts with the self-reported COVID-19 impacts. Regression analyses suggested no such interaction.

2. **p. 18; Table 2: Did the Authors check whether the entered interaction term resulted in significant change in R squared of the regression model?**

We have revised out presentation of the regression analysis to include a model selection procedure that assesses change in model fit from adding the interaction terms. We now present more parsimonious models based on this procedure, which support substantively the same conclusions as the previously presented models. It is clear from this presentation that the introduction of the critical interaction term significantly improved model fit.

3. **Figure 1: Please, provide more precise names of the axes: ERQ R could be written as Reappraisal, while COVID-19 Factor 1 (ustandardized b) – if I understand it correctly it should be: Association between self-reported Covid-19 impact (Factor 1) on SWLS (unstandardized b).**

We have edited the labels of Figure 1 as suggested.
